# Endoscopic ultrasound-guided gastronephrostomy using a lumen-apposing metal stent

**DOI:** 10.1055/a-2318-2897

**Published:** 2024-05-29

**Authors:** Ivo Horný

**Affiliations:** 1 48378 Department of Internal Medicine, Nemocnice Strakonice, Strakonice, Czech Republic

Indications for the placement of lumen-apposing metal stents (LAMSs) are constantly evolving. In this case report, we describe an unusual approach to a left kidney abscess using this endoscopic technique.

A 79-year-old woman was admitted for fever, back pain, and weight loss. Her C-reactive protein level was 325 mg/L, her white blood cell count was 16 × 10 ^9^ , and her hemoglobin was 77 g/L. She was dehydrated and had a serum creatinine of 124 µmol/L. The patient was given antibiotics. An abdominal ultrasound revealed stage IV hydronephrosis of the left kidney and detected a new multicystic expansion in the pancreatic head. Endoscopic ultrasound then showed pyonephrosis of the left kidney and evaluated the expansion in the pancreatic head as multiple benign small cysts. Finally, abdominal computed tomography (CT) revealed a large fluid collection in the left kidney with no residual kidney tissue. The left ureter was dilated and obstructed by a 7.5-mm stone in its distal part.

Because of the anatomical situation and the patient’s poor clinical condition, an unusual therapeutic approach – EUS-guided gastronephrostomy – was chosen. The patient was intubated, and an endoscope was inserted into the stomach. The renal abscess was identified at the site of the bulky prominence on the posterior gastric wall. Then, a LAMS stent (Hot AXIOS, Boston Scientific) was inserted into the abscess cavity and placed to connect the abscess and the stomach ( Video 1 ). Finally, two double-pigtail stents were inserted into the LAMS. During the procedure, around 400 ml of purulent fluid was extracted. The patient was then observed in an intensive care unit. Within 2 days, the patient was afebrile, with substantial decreases in C-reactive protein and leucocytes levels. A follow-up CT scan of the abdomen was performed 5 days after the procedure and showed only a 3-cm residual cavity after the drainage.

Drainage of left kidney abscess by endoscopic ultrasound-guided gastronephrostomy with a lumen-apposing metal stent.Video 1

Endoscopy_UCTN_Code_TTT_1AS_2AG

## Notes

Endoscopy E-Videos https://eref.thieme.de/e-videos
*E-Videos* is an open access online section of the journal *Endoscopy* , reporting on interesting cases and new techniques in gastroenterological endoscopy. All papers include a high-quality video and are published with a Creative Commons CC-BY license. Endoscopy E-Videos qualify for HINARI discounts and waivers and eligibility is automatically checked during the submission process. We grant 100% waivers to articles whose corresponding authors are based in Group A countries and 50% waivers to those who are based in Group B countries as classified by Research4Life (see: https://www.research4life.org/access/eligibility/ ). This section has its own submission website at https://mc.manuscriptcentral.com/e-videos .

